# Brain reactivity using fMRI to insomnia stimuli in insomnia patients with discrepancy between subjective and objective sleep

**DOI:** 10.1038/s41598-021-81219-2

**Published:** 2021-01-15

**Authors:** Young-Bo Kim, Nambeom Kim, Jae Jun Lee, Seo-Eun Cho, Kyoung-Sae Na, Seung-Gul Kang

**Affiliations:** 1 Department of Neurosurgery, Gil Medical Center, Gachon University College of Medicine, Incheon, Republic of Korea; 2 Department of Biomedical Engineering Research Center, Gachon University, Incheon, Republic of Korea; 3 Gachon University College of Medicine, Incheon, Republic of Korea; 4 Department of Psychiatry, Gil Medical Center, Gachon University College of Medicine, 21, Namdong-daero 774 beon-gil, Namdong-gu, Incheon, 21565 Republic of Korea

**Keywords:** Neuroscience, Medical research

## Abstract

Subjective–objective discrepancy of sleep (SODS) might be related to the distorted perception of sleep deficit and hypersensitivity to insomnia-related stimuli. We investigated differences in brain activation to insomnia-related stimuli among insomnia patients with SODS (SODS group), insomnia patients without SODS (NOSODS group), and healthy controls (HC). Participants were evaluated for subjective and objective sleep using sleep diary and polysomnography. Functional magnetic resonance imaging was conducted during the presentation of insomnia-related (*Ins*), general anxiety-inducing (*Gen*), and neutral (*Neu*) stimuli. Brain reactivity to the contrast of *Ins* vs. *Neu* and *Gen* vs. *Neu* was compared among the SODS (n = 13), NOSODS (n = 15), and HC (n = 16) groups. In the SODS group compared to other groups, brain areas including the left fusiform, bilateral precuneus, right superior frontal gyrus, genu of corpus callosum, and bilateral anterior corona radiata showed significantly increased blood oxygen level dependent (BOLD) signal in the contrast of *Ins* vs. *Neu*. There was no brain region with significantly increased BOLD signal in the *Gen* vs. *Neu* contrast in the group comparisons. Increased brain activity to insomnia-related stimuli in several brain regions of the SODS group is likely due to these individuals being more sensitive to sleep-related threat and negative cognitive distortion toward insomnia.

## Introduction

Insomnia disorder is a group of heterogeneous conditions, with each patient presenting with varying clinical characteristics and etiologies of insomnia^1^. Various insomnia subtypes that were identified under the insomnia category (e.g., acute insomnia, psychophysiological insomnia, sleep state misperception, idiopathic insomnia) in the International Classification of Sleep Disorders (ICSD)-2^2^ were consolidated under a single diagnosis of insomnia disorder in the ICSD-3^3^ due to clinical convenience and insufficient scientific support for the insomnia subtypes. However, to understand the neurobiological mechanism of insomnia, studies on the characteristics of each subtype of insomnia are needed. Patients with sleep state misperception (SSM), also referred to as paradoxical insomnia, have a significant discrepancy between subjective and objective sleep, tending to overestimate sleep latency while underestimating total sleep time (TST) relative to objective sleep measures (e.g., sleep measurements in polysomnography [PSG])^4^.

The proportion of subjective insomnia subtypes was 9.2–26.4% among insomnia patients^5,6^, and up to 50% of people experiencing insomnia can be poor estimators of sleep^7^. According to a previous small sample study, the proportions of sleep stage percentages were similar between the SSM and objective insomnia groups, but differed from the normal subjects^8^. However, sleep continuity was not different between the SSM and normal control groups^8^. In addition, the sleep patterns of each of the three groups were consistent over the 6 nights of PSG during long-term follow up^8^. In another study, the subjective insomnia group showed greater alpha, sigma, and beta non-rapid eye movement (NREM) and lower delta electroencephalogram activity than that of the normal subjects, however, these measures did not differ between the objective insomnia group and normal subjects^9^. SSM is one of the subtypes of insomnia, but it is also a common phenomenon in chronic insomnia. Early studies of sleep perceptions among insomnia patients tended to suggest that over-estimation of sleep difficulty is a general trait of individuals experiencing insomnia. For example, a previous study compared the PSG findings and self-reports of insomnia patients, and showed that less than 20% of insomnia patients had short sleep time or long sleep latency according to the PSG^10^. In another study, insomnia patients tended to underestimate total sleep time and overestimate sleep onset latency, whereas the opposite trend was found in normal sleepers^11^. Several hypotheses to explain SSM have been proposed: (a) sleep being misperceived as wake, (b) selective attention and worry about sleep-related threats, (c) increased physiological and cortical arousal, and (d) psychological distress causing magnification of sleep disturbance^4^.

Up to 50% of insomnia becomes chronic^12^. Key factors in the cognitive model of the maintenance of insomnia include a distorted perception of sleep deficit, selective attention and monitoring for sleep-related threats, excessive negatively toned cognitive activity, etc^13^. It has been reported that cognitive distortion is more severe in SSM than in other types of insomnia^4^. Functional neuroimaging is a good methodology to investigate cognitive distortion, particularly in examining selective attention to insomnia/sleep-related threats seen in paradoxical insomnia and insomnia disorder. However, there have been few functional magnetic resonance imaging (fMRI) studies on this topic. In studies investigating brain reactivity to sleep-related pictures or words in insomniacs, results have been mixed with both significant^14,15^ and nonsignificant^16^ findings. Additionally, the studies with significant findings were inconsistent as to what brain areas showed significantly different activation^14,15^.

It is thought that the results of those previous brain imaging studies have been inconsistent due to the heterogeneous nature of insomnia. With respect to SSM specifically, minimal fMRI research has been conducted. Associations of subjective–objective discrepancy of sleep (SODS) and alterations in regional glucose metrics in patients with insomnia compared with good sleeper controls have been reported in a [^18^F]fluorodeoxyglucose positron emission tomography (FDG-PET) study^17^. However, there have been no other brain imaging studies to reveal the functional neurobiological underpinnings of insomnia with SSM.

The aims of this study were (1) to investigate the differences in brain reactivity to insomnia-related negative stimuli among insomnia patients with SODS (SODS group), insomnia patients without SODS (NOSODS group), and healthy controls (HC); (2) to investigate differences in brain reactivity to general anxiety-inducing stimuli among these three groups; and (3) to identify the brain areas that show differences in brain reactivity to insomnia-related negative stimuli between insomnia disorder (ID) generally (SODS + NOSODS) and the HC group. We hypothesized that would be significantly increased brain reactivity to insomnia-related negative stimuli during fMRI in the SODS group compared to the NOSODS and control groups.

## Results

### Demographics, clinical characteristics, and polysomnographic data

Among the 68 recruited participants in the insomnia group, 26 were excluded from the study at the baseline screening for the following reasons: metal material in the body (*n* = 7), Insomnia Severity Index (ISI) score < 15 (n = 4), left-handedness (*n* = 1), suspected obstructive sleep apnea before PSG (*n* = 7), suspected depressive disorder (*n* = 6), and excessive caffeine use (*n* = 1). Between baseline screening and the end of the study, 14 patients were excluded. One subject was excluded due to an Apnea–Hypopnea Index (AHI) ≥ 15 after PSG and another subject was excluded due to a structural abnormality in the brain after brain magnetic resonance imaging (MRI) scanning. The remaining 12 subjects were excluded at some point between baseline screening and MRI scanning due to withdrawal of consent (*n* = 11) and loss of contact (*n* = 1). Among the 37 recruited HC participants, 11 were excluded from the study at baseline screening for the following reasons: metal material in the body (*n* = 6), suspected insomnia from the questionnaires and interviews (*n* = 3), and left-handedness (*n* = 2). Between baseline screening and the end of the study, 10 subjects were excluded. Two subjects were excluded due to sleep efficiency < 70% after PSG and eight subjects were excluded due to withdrawal of consent at some point between baseline screening and MRI scanning. Among the participants, 28 patients with insomnia and 16 HCs were ultimately included in the present study.

Participants with insomnia disorder were divided into the SODS (*n* = 13) and NOSODS (*n* = 15) groups according to the criteria described in the Methods section. The demographic and clinical characteristics of the SODS, NOSODS, and HC groups are presented in Supplementary Table 1. The three groups differed significantly in age (*p* = 0.001) and years of education (*p* = 0.001). However, there was no significant difference in sex among these three groups, and there was no difference in age and sex between the ID and HC groups. The mean duration of insomnia was 96.9 months in SODS, 101.7 months in NOSODS, and 100.1 months for the total insomnia group. There was no significant difference in the duration of insomnia or total ISI and Pittsburgh Sleep Quality Index (PSQI) scores at screening between the SODS and NOSODS groups. However, the total ISI and PSQI scores at screening in the ID group were higher than those in the HC group (*p* < 0.001).

The average TST (258.2 vs. 351.2 vs. 425.7 min) and sleep efficiency (52.7 vs. 76.6 vs. 94.0%) in the sleep diary were significantly different among the three groups (SODS vs. NOSODS vs. HC, respectively) (*p* < 0.001, Supplementary Table 1). The PSG results showed a significant difference in sleep efficiency among the three groups (*p* = 0.018). However, there was no significant difference in TST, sleep latency, apnea–hypopnea index, or periodic limb movements during sleep index among the three groups (*p* > 0.05).

### Blood oxygen level dependent (BOLD) signal comparisons to the contrast of *Ins* vs. *Neu* among groups

**Figure 1:**
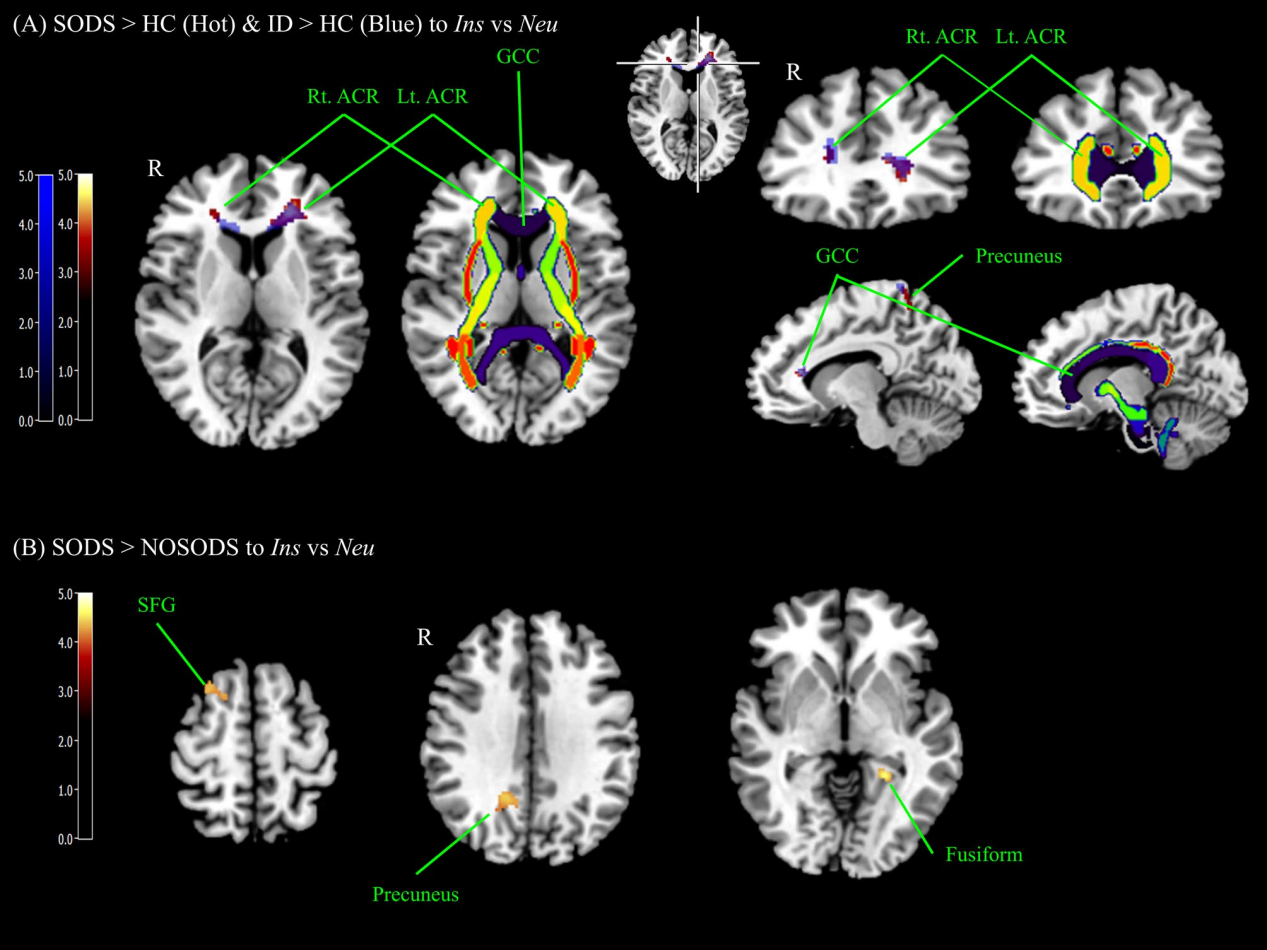
Brain areas showing significantly increased BOLD signals in the contrast of *Ins* vs. *Neu* among groups. (**A**) Brain areas showing increased BOLD signals to the contrast of *Ins* vs. *Neu* among groups (SODS vs. NOSODS vs. HC; ID vs. HC). Brain areas showing increased BOLD signals to the contrast of *Ins* vs. *Neu* in SODS and ID compared to HC group were GCC and bilateral ACR and precuneus. There were no significant areas in NOSODS compared to the HC group. (**B**) In the SODS group compared to the NOSODS group, right SFG, left fusiform, and right precuneus showed a statistically significant difference in the contrast of *Ins* vs. *Neu*. The statistical threshold was voxel-wise uncorrected *p* < 0.001 with cluster-wise FWE corrected *p* < 0.05 (141 voxels). *SODS* insomnia with subjective–objective discrepancy of sleep group, *NOSODS* insomnia without subjective–objective discrepancy of sleep group, *HC* healthy control, *ID* insomnia disorder group (SODS + NOSODS), *Ins* insomnia item, *Neu* neutral item, *GCC* genu of corpus callosum, *ACR* anterior corona radiate, *SFG* superior frontal gyrus, *FEW* family wise error.

In the SODS and ID groups compared to the HC group to the contrast of *Ins* vs. *Neu*, brain areas including the genu of the corpus callosum (GCC), bilateral anterior corona radiata (ACR), and left precuneus showed significantly increased BOLD signal. Conversely, there was no brain region with a significant difference in the NOSODS compared to the HC groups. In the SODS group compared to the NOSODS group, brain areas such as right superior frontal gyrus (SFG), right precuneus, and left fusiform showed significantly increased BOLD signal (Fig. 1, Table 1). Figures showing all the slices of the GCC and both ACR can be found in Supplementary Fig. 1.

**Table 1:** Group comparison results exhibiting significantly increased BOLD signals in the *Ins* vs. *Neu* and *Gen* vs. *Neu* contrasts.

Group comparison	Region	*Ins*–*Neu*					*Gen*–*Neu*
		Peak MNI coordinate (mm)			Peak Z score	Extents (K_E_)	
		X	y	z			
SODS > HC	GCC and Lt. ACR	− 22	36	− 4	4.74	512	NS
	Rt. ACR	20	28	6	4.00	162	
	Lt. Precuneus	− 18	− 40	58	3.83	405	
NOSODS > HC	NS						NS
SODS > NOSODS	Lt. Fusiform	− 24	− 44	− 14	4.30	161	NS
	Rt. Precuneus	14	− 54	24	4.16	157	
	Rt. SFG	18	14	48	3.86	244	
ID > HC	GCC Lt. ACR	− 22	36	− 4	4.99	491	NS
	Rt. ACR	20	32	8	4.62	261	
	Lt. Precuneus	− 18	− 40	58	4.04	273	

The statistical threshold was voxel-wise uncorrected *p* < 0.001 with cluster-wise FWE corrected *p* < 0.05 (141 voxels). FWE corrected cluster size was calculated using 3dClusSim. x, y, z are coordinates of maximum activated brain region in MNI (Montreal Neurologic Institute) stereotactic standard brain template.

*SODS* insomnia with subjective–objective discrepancy of sleep, *NOSODS* insomnia without subjective–objective discrepancy of sleep, *HC* healthy control, *NS* no significant finding, *ID* insomnia disorder (SODS + NOSODS), *Ins* insomnia item, *Neu* neutral item, *Gen* general item, *GCC* genu of corpus callosum, *ACR* anterior corona radiate, *SFG* superior frontal gyrus, *FEW* family wise error.

The regression analysis examining the association of SODS, using the TST (TST from the sleep diary minus TST from PSG), and the BOLD response revealed a statistically significant association between the BOLD response of the left medial frontal cortex and TST when using the criteria of voxel-wise uncorrected *p* < 0.001 with a cluster size of 30 (Supplementary Fig. 2 and Supplementary Table 2). However, this association disappeared when the threshold of statistical significance was set at voxel-wise uncorrected *p* < 0.001 with a cluster-wise corrected *p* < 0.05 (*K* = 141).

### BOLD signal comparisons to the contrast of *Gen* vs. *Neu* among groups

In the pairwise group comparisons to the contrast of *Gen* vs. *Neu*, there was no significant difference in the comparison of SODS > HC, NOSODS > HC, and SODS > NOSODS groups. Further, there was no significant difference in the comparison of and ID > HC groups. (Table 1).

### Correlation among the clinical data and BOLD signals in the ROIs

**Figure 2:**
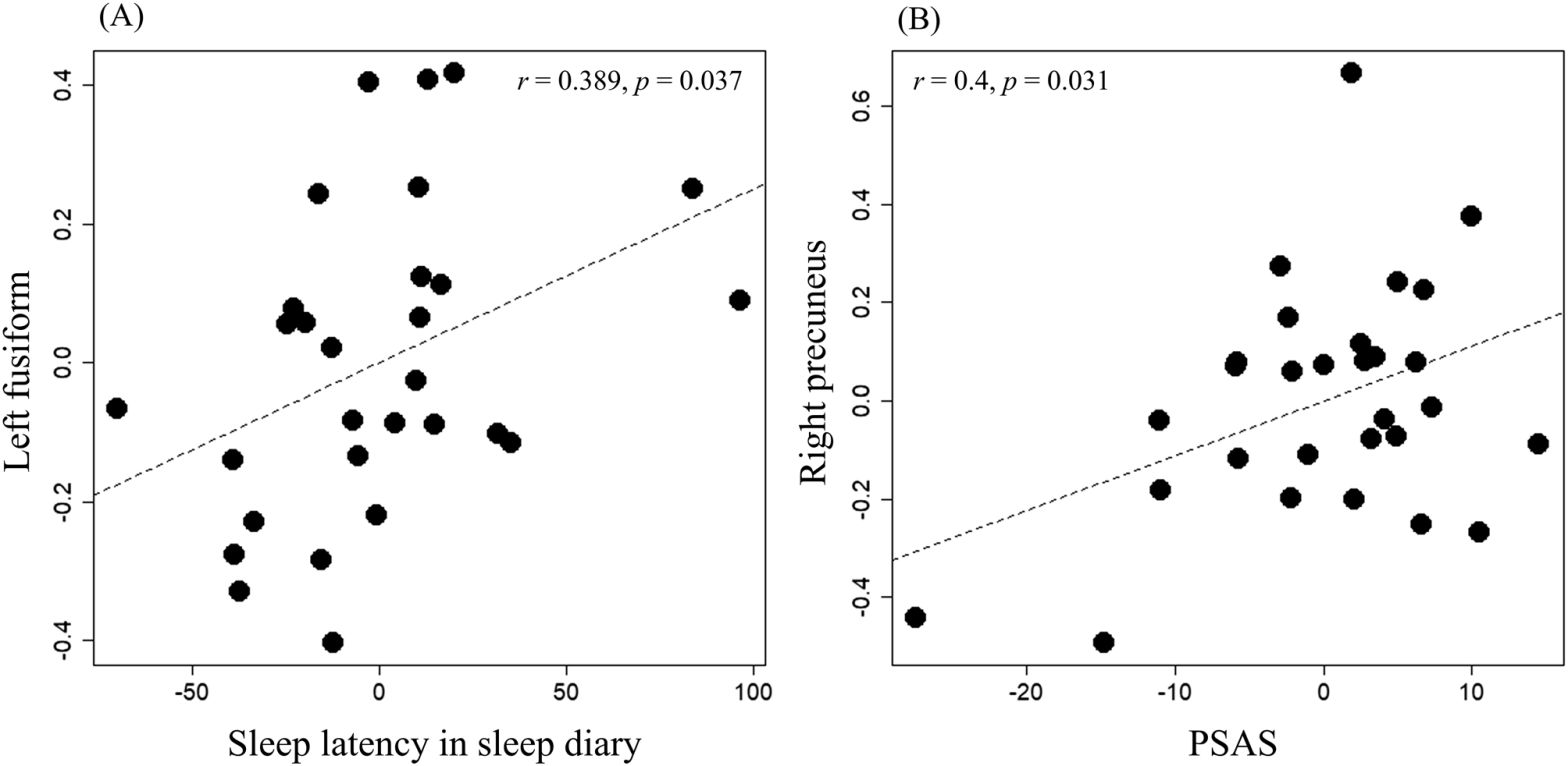
Partial correlations between clinical data and BOLD activation in the ROIs. The partial correlation (**A**) between sleep latency from the sleep diary and BOLD signal activation in the left fusiform (*r* = 0.389, *p* = 0.037) and (**B**) between PSAS and in the right precuneus (*r* = 0.4, *p* = 0.031) were statistically significant, respectively. *BOLD* blood oxygen level dependent, *ROI* region of interest, *PSAS* pre-sleep arousal scale.

Six ROIs that showed significantly different brain activation among groups in voxel-wise whole-brain analyses were selected: bilateral ACR, bilateral precuneus, right SFG, and left fusiform. As the GCC cluster was connected to the left ACR cluster, we did not perform an analysis for the GCC area. In 28 patients with insomnia, we performed partial correlation analyses among clinical data (total scores of ISI, Pre-Sleep Arousal Scale [PSAS], and sleep latency and TST of the sleep diary) and BOLD signal of the ROIs, controlling for age, sex, and years of education. Two areas showed statistically significant correlations between their BOLD signals and clinical data: sleep latency of the sleep diary and BOLD signal activation in the left fusiform (*r* = 0.389, *p* = 0.037) and PSAS score and BOLD signal activation in the right precuneus (*r* = 0.4, *p* = 0.031; Table 2, Fig. 2). There was no other significant brain area that had a significant correlation between BOLD signal activation and clinical data.

**Table 2:** Partial correlation results between clinical data and ROI BOLD activation to the *Ins* vs. *Neu* contrast.

Clinical data	Statistics	SODS > HC			SODS > NOSODS		
		Lt. ACR	Rt. ACR	Lt. Precuneus	Lt. Fusiform	Rt. Precuneus	Rt. SFG
ISI total score	*R*	− 0.202	− 0.165	0.146	− 0.116	0.039	0.111
	*p* value	0.29	0.39	0.45	0.55	0.84	0.56
PSAS total score	*r*	0.0748	0.14	0.062	0.14	0.4	0.136
	*p* value	0.7	0.47	0.75	0.47	**0.031**	0.48
Sleep latency in sleep diary	*r*	0.215	− 0.074	0.031	0.389	0.207	− 0.22
	*p* value	0.26	0.7	0.87	**0.037**	0.28	0.25
TST in sleep diary	*r*	0.105	0.156	0.181	0.17	− 0.045	0.185
	*p* value	0.59	0.42	0.35	0.38	0.82	0.34

*ROI* region of interest, *BOLD* blood oxygen level dependent, *Ins* insomnia item, *Neu* neutral item, *SODS* insomnia with subjective–objective discrepancy of sleep, *NOSODS* insomnia without subjective–objective discrepancy of sleep, *HC* healthy control, *ACR* anterior corona radiate, *SFG* superior frontal gyrus, *ISI* Insomnia Severity Index, *PSAS* Pre-Sleep Arousal Scale, *TST* total sleep time.

Significant results are highlighted in bold.

## Discussion

In this study, we found that the SODS and ID groups showed higher brain reactivity to insomnia-related negative stimuli in the GCC, bilateral ACR, and left precuneus regions compared to HC. The SODS group showed higher brain reactivity in the right SFG, precuneus, and left fusiform compared to the NOSODS group. Conversely, for the general anxiety-inducing stimuli, neither the SODS nor NOSODS groups showed increased brain reactivity compared to HC.

Previous studies have shown that individuals with SSM are sensitive to insomnia-related cues^4^. The tendency toward selective attention and monitoring has been proposed to contribute to the misperception of sleep^4^. Selective attention and monitoring can be roughly categorized into external monitoring (e.g., clock sound at night) and internal monitoring (e.g., bodily sensations of signs of poor sleep)^4^. When patients detect these cues, they misinterpret them as indicative of a threat to sound sleep^18^. It is believed that such a threat overactivates the brain, which can cause hyperarousal, which in turn can cause arousal and insomnia. This study is important in being able to test this theory directly through fMRI.

Few neuroimaging studies of sleep misperception have been performed. Kay et al. reported a significant group (primary insomnia and good sleeper) by sleep latency discrepancy interaction for regional cerebral metabolic rate for glucose (rCMR_glc_)^17^, finding a significant positive correlation between sleep latency discrepancy (self-reported minus PSG-measured sleep latency) and rCMR_glc_ in the middle/posterior cingulate and right anterior insula during NREM sleep in primary insomnia and a significant negative correlation between sleep latency discrepancy and rCMR_glc_ in the left anterior and middle/posterior cingulate cortex and right anterior insula^17^.

Three fMRI studies using insomnia-related stimuli have been previously conducted. Baglioni et al. showed increased amygdala reactivity to insomnia-specific stimuli (e.g., pictures of lying awake in bed at night) in patients with chronic insomnia^14^. Kim et al. reported hyper-responses of the precentral cortex, prefrontal cortex, and default mode network to insomnia-related pictures in psychophysiological insomnia and normalization of hyper-responses after cognitive behavioral therapy for insomnia (CBT-I)^15^. In addition, Kim et al. reported that in an fMRI study before and after CBT-I in patients with psychophysiological insomnia, brain activity in the left middle temporal and occipital areas decreased after treatment^19^. Spiegelhalder et al., in order to further the study of Baglioni et al., collected fMRI during the presentation of sleep-related and control words with positive and negative valence^16^. Contrary to their hypothesis, the whole-brain analysis of sleep-related vs. control words contrast did not show any significant cluster of voxels in a between-group comparison^16^.

In this study, the SODS group showed increased brain activity to the contrast of *Ins* vs. *Neu* in the GCC, bilateral ACR, bilateral precuneus, right SFG, and left fusiform compared to the other groups. The corpus callosum is one of the largest white matter structures, and the main function of the GCC is communication between the frontal lobes of the left and right hemispheres and it also plays a role in cognition^20^. The role of the corpus callosum in sleep has not been clarified, but in a previous case report, it was speculated that it facilitates synchronization of activity between homologous regions in the two hemispheres^21^. In a resting-state fMRI study, connectivity in the primary insomnia group compared to healthy controls was stronger between the bilateral superior parietal lobe and right splenium of the corpus callosum^22^. A tract-based spatial statistics diffusion tensor imaging (DTI) study of insomnia demonstrated a reduced fractional anisotropy (FA) value in the corpus callosum^23^. ACR is also reported to have a reduced FA value in patients with primary insomnia^23^ and people with poor sleep quality^24^. Combining the results of these DTI studies^23,24^ with the current results, the increased BOLD signal of the SODS group might be attributed to a disruption of the white matter track of the GCC and ACR in insomnia. In this study, the NOSODS group did not show a difference in BOLD signal in GCC and ACR compared to the HC group, so it seems important to subtype insomnia patients in future insomnia fMRI studies.

In addition, GCC and ACR are white matter, which is a novel finding in a task fMRI study using insomnia-related stimuli. It has been commonly thought that activation in white matter is a false representation of underlying neural activity, and as such, any increased BOLD signal in the white matter of previous fMRI studies has not been reported^25^. BOLD signal in white matter has been considered as an artifact mainly due to cardiac, respiration, and motion. Cardiac noise mainly occurs around major vessels^26,27^, which is different from the significant white matter areas in our study. In the case of respiratory artifacts, small fluctuations in end-tidal CO_2_ at a frequency range of 0–0.05 Hz occurs during normal breathing at rest, and has been significantly correlated with BOLD signal fluctuations^28^. However, the block design with a jittering interstimulus interval in our study reduces the sensitivity of respiratory artifacts^29^. With respect to head motion artifact, it occurs mainly in individual analyses, but does not seem to change the result of group analyses^30^. Recently, increasing evidence of detectable and true BOLD signal has been reported in white matter areas such as the corpus callosum^31–35^, as such, these increases in BOLD signal in white matter are no longer ignored^36^.

The SFG is an area previously reported as showing significant activation in fMRI studies in insomnia. Compared with female good sleepers, female chronic primary insomnia patients showed lower regional homogeneity (ReHo) of brain activity in the left SFG^37^. In another study, patients with primary insomnia showed decreased connectivity in regions of the right frontoparietal network, including the SFG^38^. In addition, in a resting-state fMRI study of 15 participants with insomnia and 15 controls, functional connectivity between the superior parietal lobe and SFG was lower in patients with primary insomnia^22^.

Among the significant brain areas in our study, the right precuneus and left fusiform also showed significant correlations with clinical sleep variables. In a previous PET study, the precuneus and left fusiform were reported as regions showing significant interaction between group (insomnia vs. good sleeper) and state (wake vs. NREM sleep) in relative rCMR_glc_^39^. Although the function of the precuneus is not clearly understood, it is known to perform functions related to self-consciousness, episodic memory, visuospatial ability, executive function, and consciousness^40–42^. In PET studies, the precuneus was reported to be involved in consciousness, and wake and sleep states, and to be related to the most inactive metabolism during slow wave and REM sleep and to the most active metabolism during wakefulness^41,43^. The fusiform is also an area where significant results have been reported in other fMRI studies in insomnia^15,37,44–46^. Compared to HCs, patients with primary insomnia showed higher BOLD activation in the fusiform to sleep-related pictures^15^. Compared with female good sleepers, female insomnia patients showed higher ReHo in the left fusiform gyrus^37^. Other resting-state fMRI studies also showed increased connectivity between the fusiform and other brain areas in patients with insomnia and healthy participants with insomnia^44,45^. Conversely, another study has shown reduced connectivity^46^.

It is interesting that insomnia patients did not show increased brain reactivity to general anxiety-inducing stimuli, although they did show increased brain reactivity to insomnia-related stimuli. This result suggests that insomniac patients are less responsive to general anxiety-inducing stimuli because they are selectively focused on insomnia-related stimuli and anxiety, although the possibility of false-negatives in this study cannot be excluded due to the small sample size.

Our study has several limitations. First, the sample size of our study is small, though the study is worthy as a first study on this topic. Second, since the distinction between the SODS and NOSODS groups in this study was arbitrary, in order to differentiate between insomnia phenotypes, it would be desirable to recruit participants with subjective insomnia meeting the SSM criteria of the ICSD-2^2^ and compare them with an objective insomnia group.

To the best of our knowledge, our finding of increased brain reactivity in the left precuneus, GCC, and bilateral ACR to insomnia-related stimuli in the SODS and ID groups compared to the HC group is the first. In addition, the SODS group showed increased brain activity in the left fusiform, right precuneus, and right SFG to insomnia-related stimuli compared to the NOSODS group. General anxiety-inducing stimuli did not cause any differences in brain activity among the groups. It could be said that functional neuroimaging has demonstrated that in insomnia, especially SODS, people experience selective attention to insomnia-related threats. In order to broaden the understanding of neurobiology of sleep misperception, replication of our results and further research on this subject are needed in the future.

## Materials and methods

### Participants and evaluation of sleep and clinical status

Patients with insomnia disorder and HC were recruited from the Sleep Medicine Center at Gil Medical Center in Incheon, South Korea. Recruitment was conducted through a notice posted on the bulletin board of Gil Medical Center, via physician-initiated referrals or requests, and an online advertisement. All participants were aged 18–70 years, right-handed, and screened through telephone prescreening interviews. After prescreening, board-certified psychiatrists who specialize in sleep medicine interviewed the participants to assess their eligibility based on the Structured Clinical Interview for Diagnostic and Statistical Manual of Mental Disorders, 5^th^ edition (DSM-5)^47^. Written informed consent was obtained from all participants prior to their inclusion in the study. The present study was approved by the Institutional Review Board of the Gil Medical Center. PSG and fMRI were conducted on all enrolled participants who had passed through the screening interview. Task fMRI was performed 1–2 weeks after PSG. Sleep diary information and actigraphy were also collected (described below) from the day after the PSG until fMRI scanning.

The inclusion criteria for insomnia patients were as follows: (a) meeting the diagnostic criteria for insomnia disorder in the DSM-5^48^ and insomnia lasting at least 6 months; (b) moderate to severe degree of insomnia complaint (a total score ≥ 15 on the ISI^49^ at screening); and (c) one or more of the following occurring 3 days or more per week (as determined by the sleep diary): total wake time (sleep latency + wake after sleep onset [WASO]) > 60 min, sleep latency > 45 min, or WASO > 45 min.

SODS was assessed by the difference between sleep diary-actigraphy over the course of 7–14 days. In case of loss of actigraphy data, SODS was evaluated according to the difference between sleep diary-PSG. Individuals were classified as the SODS group if two or more of the following criteria were met: the difference of TST ≥ 60 min, difference of sleep efficiency ≥ 15%, and difference of sleep latency ≥ 60 min. If these criteria were not met, individuals were assigned to the NOSODS group. These criteria, using the difference between subjective and objective sleep measures, were based on a previous study^7^.

The inclusion criteria for HC were as follows: (a) no symptoms or history of psychiatric disorders or sleep disorders including insomnia, (b) a total score < 4 on the ISI at screening, (c) average TST ≥ 6 h and sleep efficiency ≥ 85% in a sleep diary, and (d) no history of psychotropic medications or hypnotics.

The exclusion criteria for all participants were as follows: (a) suspected to have a major sleep disorder other than insomnia disorder, as determined by medical history; (b) high risk of sleep apnea based on the Korean Berlin Sleep Questionnaire^50^ or severe diurnal sleepiness (i.e., the total score of the Korean version of the Epworth Sleepiness Scale (ESS) ≥ 17)^51^; (c) evidence of a moderate or severe degree of sleep apnea (i.e., AHI ≥ 15); (d) any other sleep disorder except insomnia according to polysomnography (PSG) findings (e.g., Periodic Limb Movements in Sleep ≥ 15, or rapid eye movement [REM] sleep without atonia); (e) shift work or travel accompanied by frequent jet lag; (f) body mass index ≥ 30; (g) current or past diagnosis of other major psychiatric disorders (except insomnia disorder) based on a clinical interview; (h) severe depression symptoms based on the screening scale (Hamilton Depression Rating Scale-17 [HDRS-17] minus sleep item scores [i.e., items 4, 5, and 6] ≥ 20^52^; (i) history or presence of significant neurological or medical illnesses; (j) contraindications for 3 T MRI, such as metal implants, pacemakers, or claustrophobia; (k) lactation, pregnancy, or plans to become pregnant during the study period; and (l) structural abnormalities based on brain MRI.

All participants refrained from any psychoactive medication, including hypnotics, for at least two weeks prior to and during study participation. They also refrained from alcohol, caffeine, and daytime naps during the PSG and fMRI scanning days. All participants were interviewed using HDRS-17 and asked to respond to the Korean versions of the ISI, Epworth sleepiness scale, and Berlin Questionnaire at screening. They also completed the Korean versions of the Pittsburgh Sleep Quality Index, ISI, HDRS-17, and PSAS^53^ on the scanning date.

We declare that all experiments on human subjects were conducted in accordance with the Declaration of Helsinki, and that all procedures were understood adequately by the subjects, who also provided written informed consent. We also certify that formal approval to conduct the experiments was obtained from the Intuitional Review Board (IRB No. GDIRB2017-297) of the Gil Medical Center.

### Polysomnography and actigraphy

An overnight PSG was performed using the Embletta MPR sleep system (Natus, Pleasanton, CA, USA) to exclude participants with occult sleep disorders such as sleep apnea. Manual scoring was performed according to the American Academy of Sleep Medicine 2.4.^54^, with RemLogic 3.4.4 (Embla Systems; Kanata, ON, Canada) as the scoring platform. The PSG recordings were analyzed by well-trained technologists and the results were confirmed by a sleep-specialist medical doctor (S.G.K.). The Actiwatch 2 device (Respironics Incorporated, Murrysville, PA, USA) was used for actigraphy measurements. The actigraphy data were collected in 30-s epochs and analyzed using the default medium sensitivity threshold (40 counts per epoch) with Actiware software (version 6.0.8, Philips Respironics, Bend, OR, USA). The subjects were instructed to press the actigraphy event marker when going to bed at night and waking up in the morning. The rest intervals and sleep latency were defined automatically by the Actiware software using the default algorithm. In our study, the major rest interval per day (nocturnal sleep period) was used.

### Development of the experimental paradigm

In order to develop a sentence task to be shown during fMRI, a valence test was conducted on separate subjects before the start of the brain imaging study. We developed 50 sentences for each of the following three categories: (a) insomnia-related negative sentences that may cause anxiety and worries related to sleep/insomnia (insomnia-related stimuli, insomnia items, *Ins*); (b) sentences that can usually trigger anxiety and worry to ordinary people (general anxiety-inducing stimuli, general anxiety items, *Gen*); (c) neutral sentences that will not cause anxiety or worry (neutral stimuli, neutral items, *Neu*). A valence test was conducted with pen and paper on the level of anxiety or worry for these sentences in 28 patients with insomnia and 54 healthy people. They were instructed to express anxiety or worry using a Likert scale with a score of 0 (completely free of anxiety and worries) to 10 (very severe anxiety or worry). Insomnia items were selected if the sentence caused an average of 7 or more on anxiety in patients with insomnia and general items were selected if the sentence had an average of 7 or more on anxiety in both the insomnia and healthy groups. Neutral items were selected if the sentence had an average of 1 or less on anxiety in both groups. Finally, 15 sentences for each category were selected (Supplementary Methods S1).

### MRI data acquisition

A 3-T whole-body Siemens scanner (TrioTim Syngo; Siemens, Erlangen, Germany) was used for functional image acquisition with an interleaved T2*-weighted EPI gradient echo sequence (repetition time [TR], 2000 ms; echo time [TE], 28 ms; flip angle, 90°; slice thickness, 3.5 mm; in-plane resolution, 3 × 3 mm; field of view, 220 mm; matrix size, 64 × 64) with a 12-channel birdcage head coil. For each participant, two runs were acquired, and each run had 230 functional volumes. After fMRI, an anatomical image was acquired using a high, T1-weighted, 3D gradient echo pulse sequenced with magnetization prepared rapid gradient echo (TR/TE/inversion time [TI]/Flip angle = 1900 ms/3.3 ms/900 m/9°; slice thickness, 1.0 mm; in-plane resolution, 0.5 × 0.5 mm; field of view, 250 mm; matrix size, 416 × 512). The total duration of the experiment was approximately 40 min.

### fMRI experimental procedure

A sentence task was developed to be shown during fMRI and was assessed with a valence test prior to this study. Before fMRI, the participants listened to a detailed description of the task and learned the task thoroughly before scanning. The fMRI experiment had three blocks consisting of insomnia item (*Ins*), general item (*Gen*), and neutral item (*Neu*) trials that were randomly intermixed across the run. Each run had four blocks of *Ins*, *Gen*, and *Neu*. A cross sign was presented for 1 s preceding each block. In each block, an item from one of the categories (*Ins*, *Gen*, or *Neu*) was presented for 6 s during the stimulus period, followed by a cross sign for 6 s, and then a 6 s response period. During the response period, participants were asked to press one of four buttons according to the degree of anxiety they felt during the stimulus period. The degree of anxiety was categorized into four levels: no, mild, moderate, and severe anxiety. All response scores and reaction times were measured. After finishing each block, a dot sign was presented during the intertrial interval, which was jittered between 12 and 20 s. The total duration of the run was approximately 20 min. The fMRI stimulus was presented using DMDX software (http://www.u.arizona.edu/~kforster/dmdx/dmdx.htm) (Supplementary Fig. 3A).

### Statistical analysis of clinical data

The demographic and clinical data of the groups were compared using Student’s *t-*test or analysis of variance (ANOVA) and a chi-squared test. Statistical analysis was performed using SPSS for Windows ver. 23 (IBM Corp., Armonk, NY, USA), with a two-sided significance level of *p* < 0.05.

### fMRI preprocessing and analysis

fMRI data were analyzed using SPM12 (Wellcome Department of Cognitive Neurology, London, UK). The first six-volume images were discarded from the analysis in all runs to eliminate the non-equilibrium effect. The slice timing difference was corrected, and the center of the image was relocated near the anterior commissure. The volume images were realigned to the first image in the time series to correct for between-scan rigid body motion. After realignment, the volume images were co-registered to the T1-weighted images and normalized to the Montreal Neurological Institute space utilizing a transformation matrix developed from the T1 image segmentation, using methods described previously^19^. After normalization, the volume images were spatially smoothed with an 8-mm full width at half maximum isotropic Gaussian kernel to adjust residual between-participant variability as well as to increase statistical sensitivity. The resulting fMRI volume images were high-pass-filtered with a cutoff time of 128 s to remove low-frequency drifts. A voxel-based general linear model was applied to estimate the parameters, along with six motion parameters as covariates of no interest. In the subject-level analysis, three regressors were used to model *Ins*, *Gen*, and *Neu* stimuli as the conditions of interest. The response and cross sign were also modeled to reduce the residuals. Then, the design matrix was temporally convolved with a canonical hemodynamic response function to achieve a better fit. Contrast images associated with the conditions of interest (i.e., *Ins*, *Gen*, and *Neu* stimuli) were acquired for group-level analysis***.***

The group-level analysis was conducted using a factorial ANOVA design with covariates (i.e., age, sex, and years of education) to compare the differences among groups (i.e., SODS, NOSODS, and HC) and between groups (i.e., ID and HC) to the contrast of *Ins* vs. *Neu* and *Gen* vs. *Neu*. The flow of the intergroup analysis is shown in Supplementary Fig. 3B. The threshold of statistical significance was set at voxel-wise uncorrected *p* < 0.001 with cluster-wise corrected *p* < 0.05 for all analyses. The probability of false-positive clusters was estimated using 3dClustSim implemented in AFNI (https://afni.nimh.nih.gov). Regression analysis was also performed to investigate the association between the BOLD response (*Ins*–*Neu*) and measures of SODS (the difference in TST and sleep efficiency between the sleep diary and PSG). In the regression analysis, the BOLD response was modelled as a response variable, the two continuous SODS variables as explanatory variables, and three covariates as nuisance variables (age, gender, education). To estimate the smoothness of the fMRI data, we first saved the residuals while we performed SPM estimation for each subject and concatenated the time-series of residuals to 4D. Then, we estimated smoothness using 3dFWHMx. Finally, the spatial autocorrelation function parameters were calculated using the average of the smoothing estimates for each individual and used as an input for 3dclustsim in the second-level analysis. The locations of the peak z-values were reported using the automated anatomical labeling (http://www.gin.cnrs.fr/AAL2) toolbox. Visualization of fMRI results was performed using Vinci (http://vinci.sf.mpg.de).

### Region of interest (ROI) analysis

To explore the association between the clinical data (total ISI and PSAS scores and sleep latency and TST in the sleep diary) and BOLD signal change in the ROI, subsequent ROI analyses were conducted by SPM-driven ROI analysis. ROI masks were extracted from all clusters showing statistical significance in the voxel-wise comparison of SODS > HC and SODS > NOSODS in the contrast of *Ins* vs. *Neu*. Then, the β-value difference was calculated by subtracting the β-values of *Neu* from the β-values of *Ins*. Mean BOLD signals were calculated by averaging the β-values difference to conduct partial correlations with clinical data. To calculate the partial correlations between the clinical data and BOLD signals, we performed multiple regressions with the clinical data and mean BOLD signals as response variables and age, sex, and years of education as explanatory variables. After conducting ordinary least squares estimation, residuals were calculated by subtracting predicted values from the clinical data and mean BOLD signal data. Using the residuals acquired from the clinical data and the mean BOLD signal, the Pearson’s correlation coefficient was calculated between residuals of the clinical data and residuals of the BOLD signal.

## Supplementary Information

Supplementary Figure 1.

Supplementary Figure 2.

Supplementary Figure 3a.

Supplementary Figure 3b.

Supplementary Information.
