# Supplementary figures and images for: Brain reactivity using fMRI to insomnia stimuli in insomnia patients with discrepancy between subjective and objective sleep
Source: Sci Rep. 2021 Jan 15;11:1592. doi: 10.1038/s41598-021-81219-2 (PMC7810854; doi:10.1038/s41598-021-81219-2)

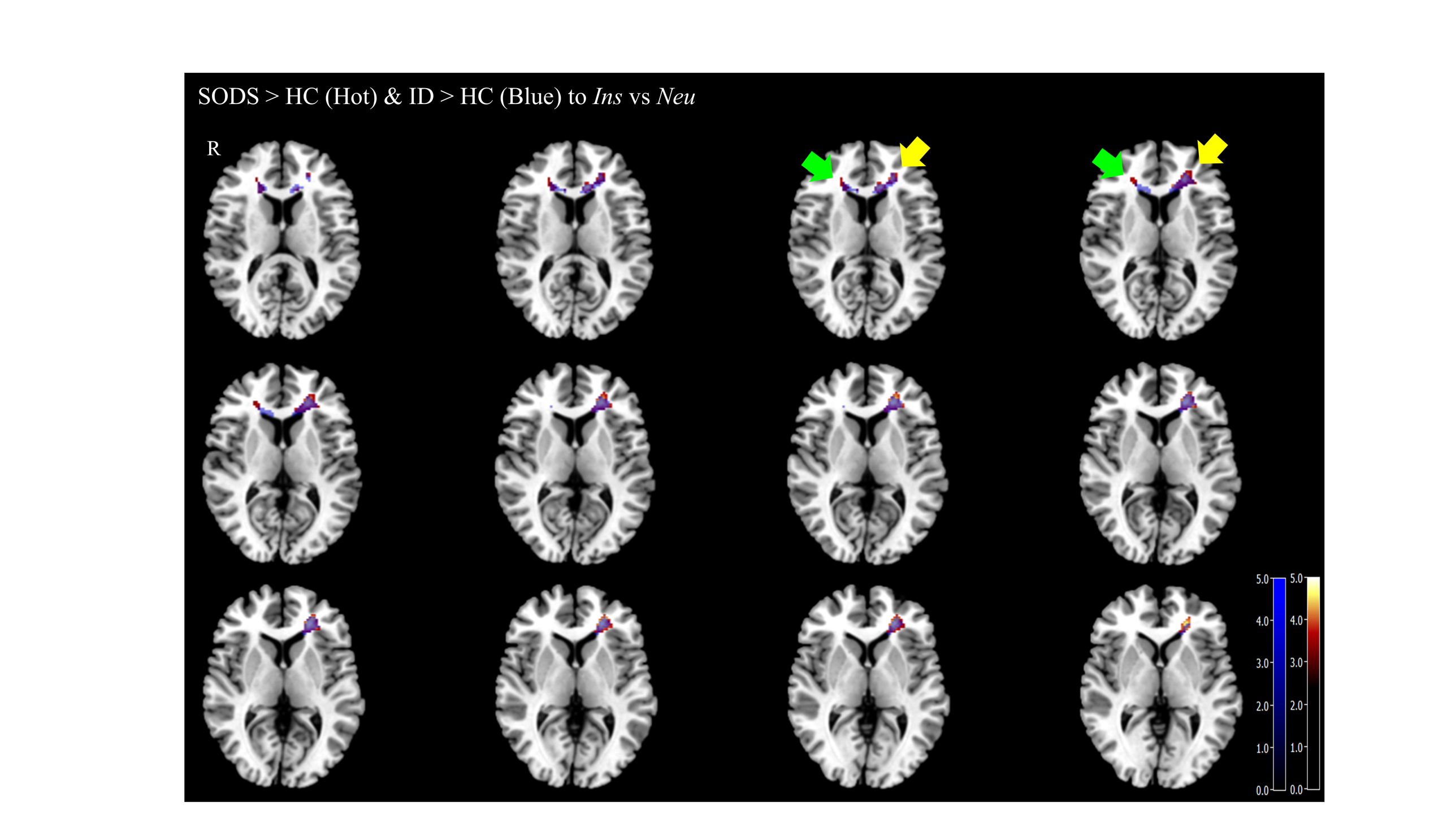

Supplement: Supplementary file 1 — Supplementary Figure 1. [file 41598_2021_81219_MOESM1_ESM.tif]

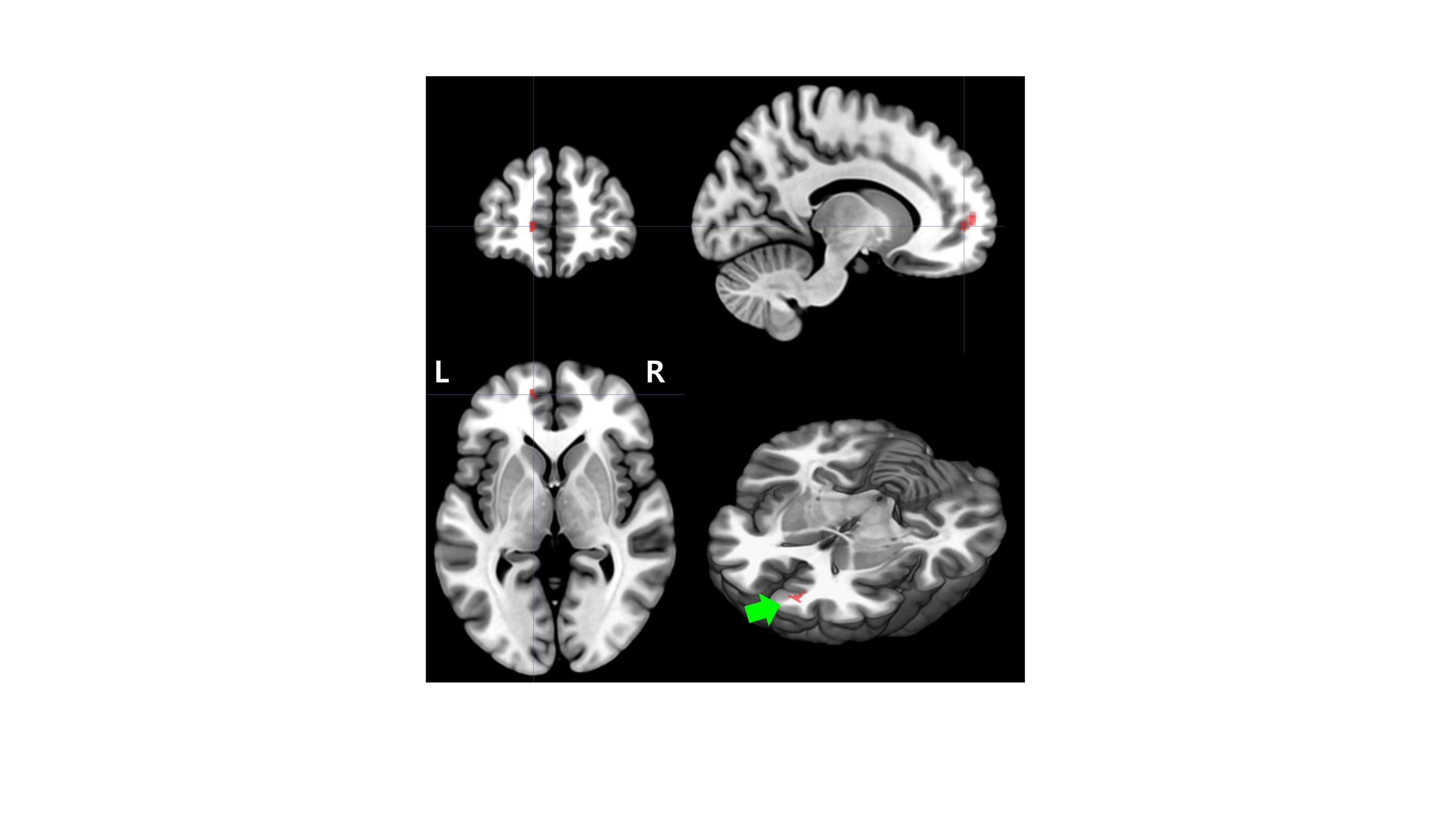

Supplement: Supplementary file 2 — Supplementary Figure 2. [file 41598_2021_81219_MOESM2_ESM.tif]

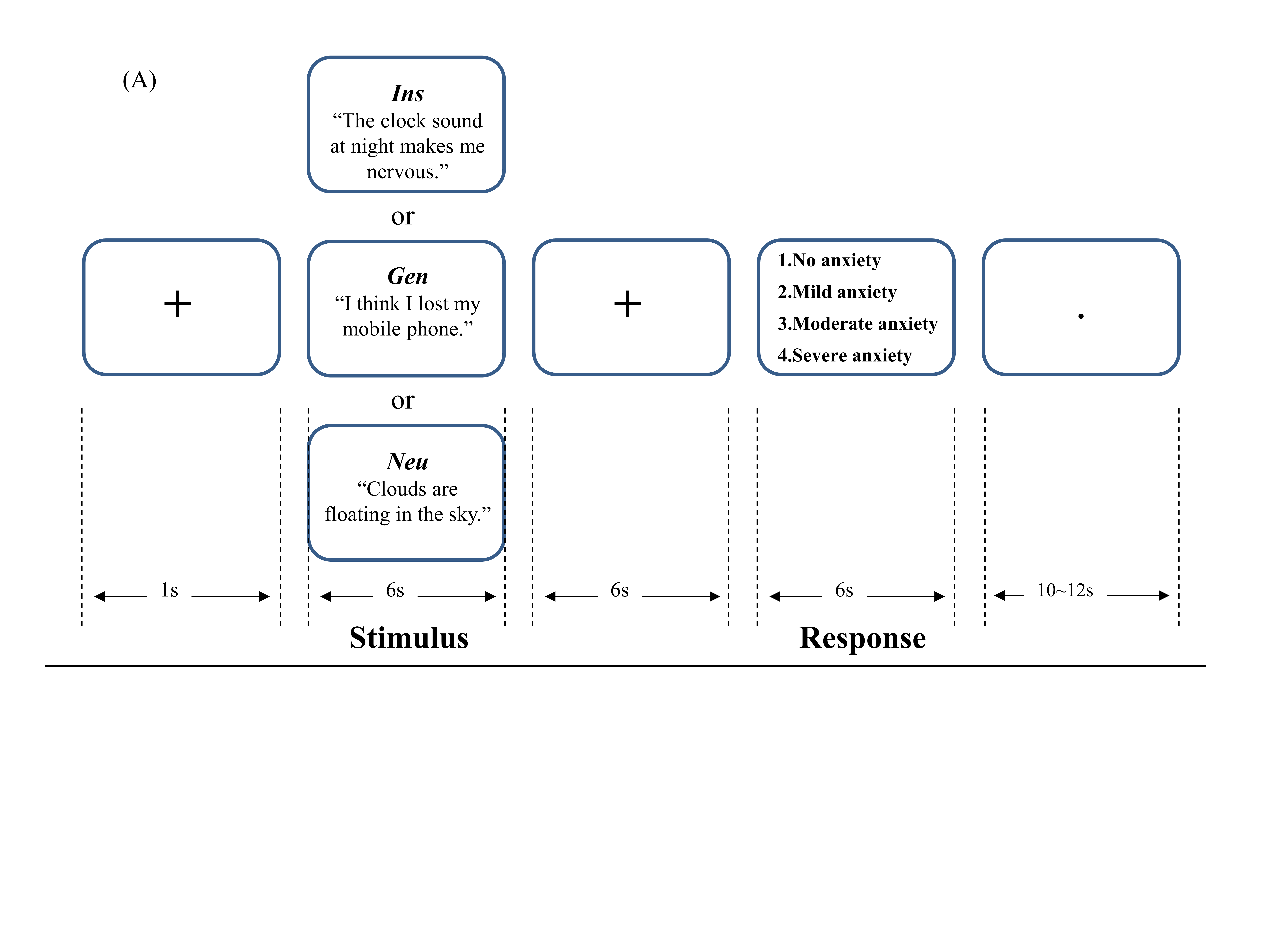

Supplement: Supplementary file 3 — Supplementary Figure 3a. [file 41598_2021_81219_MOESM3_ESM.tif]

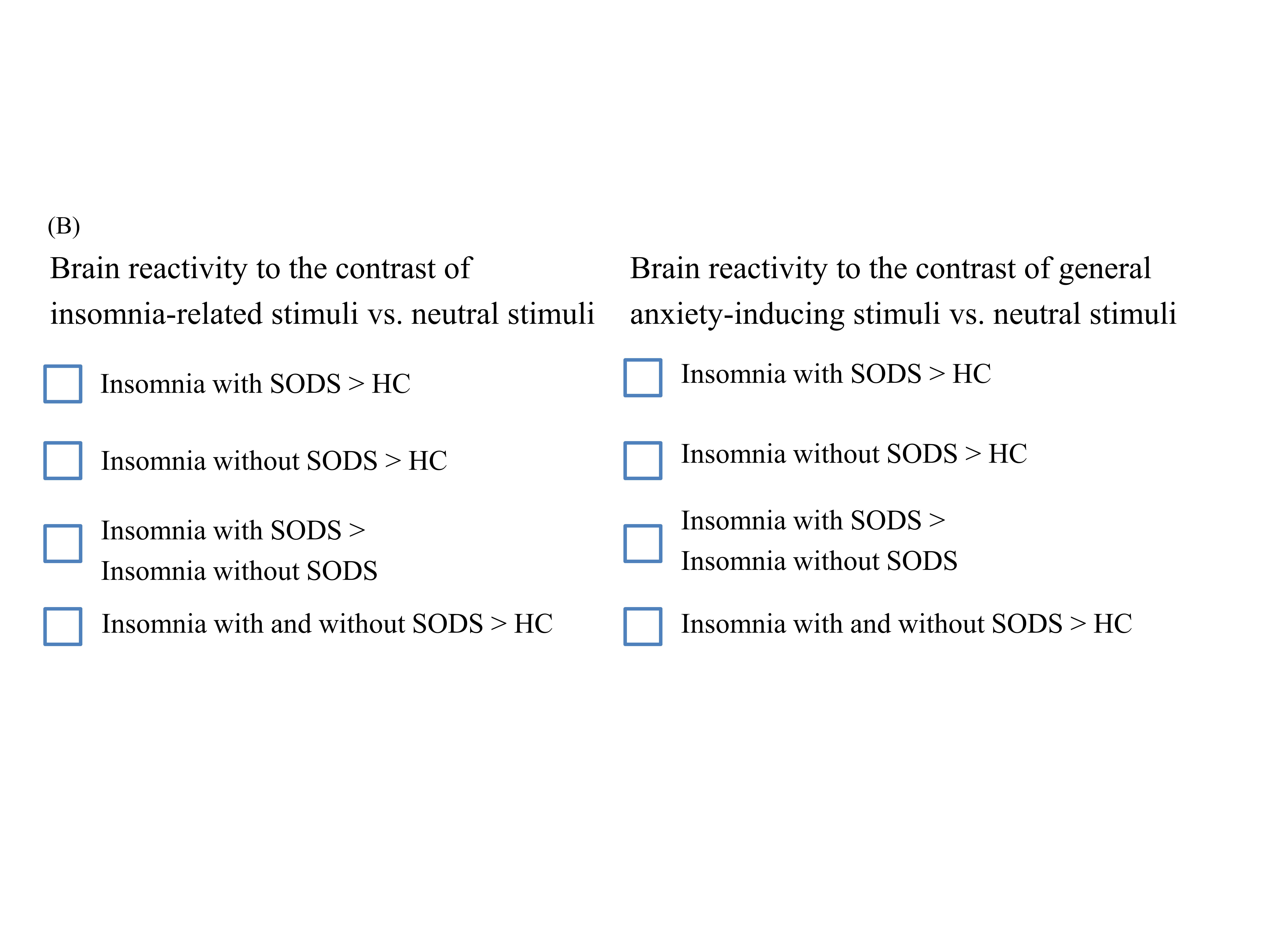

Supplement: Supplementary file 4 — Supplementary Figure 3b. [file 41598_2021_81219_MOESM4_ESM.tif]
