# Supplementary material for: Brain reactivity using fMRI to insomnia stimuli in insomnia patients with discrepancy between subjective and objective sleep
Source: Sci Rep. 2021 Jan 15;11:1592. doi: 10.1038/s41598-021-81219-2 (PMC7810854; doi:10.1038/s41598-021-81219-2)

**Brain reactivity using fMRI to insomnia stimuli in insomnia patients with discrepancy between subjective and objective sleep**

Young-Bo Kim ^1,*^, Nambeom Kim^2,*^, Jae Jun Lee^3^,

Seo-Eun Cho^4^, Kyoung-Sae Na^4^, Seung-Gul Kang^4,†^

*^1^ Department of Neurosurgery, Gachon University Gil Hospital, Incheon, Republic of Korea*

*^2^ Department of Biomedical Engineering Research Center, Gachon University, Incheon, Republic of Korea*

*^3^ Gachon University College of Medicine, Incheon, Republic of Korea*

*^4^ Department of Psychiatry, Gil Medical Center, Gachon University College of Medicine, Incheon, Republic of Korea*

^*^ Co-first authors contributed equally to this work.

^†^Corresponding author: Seung-Gul Kang, MD, PhD; Department of Psychiatry, Gil Medical Center, Gachon University, College of Medicine, 21, Namdong-daero 774 beon-gil, Namdong-gu, Incheon, 21565, Republic of Korea

Tel: +82-32-458-2818

**Supplementary Methods**

1. **The sentence stimuli used in the functional magnetic resonance imaging experiment**
2. **Insomnia items**
3. The clock sound at night makes me nervous.
4. If I can't sleep, I won't be able to work tomorrow.
5. A train of thoughts keeps me awake.
6. If I can't sleep every day, I will get sick.
7. I made every effort, but ultimately, I couldn't sleep.
8. If I can't sleep, I won't have any energy tomorrow.
9. It's nearly time to wake up, but I still can't sleep.
10. My memory is worsening because of insomnia.
11. I am afraid that I can't sleep again tonight.
12. I can't sleep because of useless worries.
13. I am afraid that insomnia is unlikely to be cured.
14. I am annoyed because I can't sleep.
15. I cannot concentrate due to insomnia.
16. I can't control my sleep.
17. I can't sleep due to noise in the living room.
18. **General items**
19. I think I lost my mobile phone.
20. I injured my leg in a car accident.
21. I did not study for tomorrow’s exam.
22. My spouse seems to be having an affair.
23. My child was kidnapped.
24. North Korea launched a missile.
25. A burglar broke into my house.
26. I suffered a serious loss in my investment.
27. My spouse became unemployed.
28. My child failed to pass the college entrance exam.
29. My favorite clothes are torn.
30. I was notified of an unexpected traffic violation fine.
31. Someone smokes without asking for my approval.
32. I have an important contract, but I am stuck in traffic.
33. My important delivery has been lost.
34. **Neutral items**
35. Clouds are floating in the sky.
36. Spring came and forsythia bloomed.
37. Watermelon is delicious in the hot summer.
38. The ginkgo leaves turned yellow.
39. The street lights are bright.
40. There are dishes on the table.
41. My mother washes cabbage in water.
42. The wind blows through the window.
43. A child plays a game on a mobile phone.
44. A woman listens to the radio with earphones.
45. Four family members eat at the table.
46. Students go to school with their friends.
47. Students are playing soccer on the playground.
48. People are jogging in the park.
49. A woman is walking with two dogs.

**Supplementary Tables**

| **Supplementary Table 1. Participants’ demographic and clinical data at baseline and sleep diary and polysomnographic data** | | | | | | | | |
| --- | --- | --- | --- | --- | --- | --- | --- | --- |
| Variables | | Insomnia disorder (*n*=28) | | HC (*n*=16) |  | Insomnia vs. HC | SODS vs. NOSODS vs. HC | |
|  |  | SODS (*n*=13) | NOSODS (*n*=15) |  |  |  |  |  |
|  |  | Mean  (SD or %) | Mean  (SD or %) | Mean  (SD or %) |  | *p* | *p* | *Significant difference after post hoc analysis^¶^* |
| Age, years | | 57.8 (8.7) | 40.3 (13.8) | 41.3 (14.7) | 0.125 | | 0.001 | SODS vs. HC  SODS vs. NOSODS |
| Sex, female | | 9 (69.2%) | 11 (73.3%) | 11 (68.8%) | 0.851 | | 1.000 |  |
| Education, years | | 11.9 (3.8) | 14.4 (3.3) | 15.8 (2.3) | 0.007 | | 0.007 | SODS vs. HC |
| Duration of insomnia disorder, months | | 96.9 (90.5) | 101.7 (125.3) | ― |  | |  |  |
| ISI score (at screening) | | 20.5 (3.3) | 20.3 (4.5) | 1.2 (1.4) | <0.001 | | <0.001 | SODS vs. HC  NOSODS vs. HC |
| PSQI (at screening) | |  |  |  |  | |  |  |
|  | Total score | 14.7 (3.0) | 13.2 (2.1) | 2.8 (1.4) | <0.001 | | <0.001 | SODS vs. HC  NOSODS vs. HC |
|  | TST, min | 267.7 (91.2) | 294.0 (85.8) | 441.3 (61.7) | <0.001 | | <0.001 | SODS vs. HC  NOSODS vs. HC |
|  | Sleep efficiency, % | 61.6 (23.6) | 74.1 (22.3) | 95.1 (7.9) | <0.001 | | <0.001 | SODS vs. HC  NOSODS vs. HC |
| PSAS (on scanning date) | | 46.0 (6.4) | 41.6 (11.4) | 18.6 (3.7) | <0.001 | | <0.001 | SODS vs. HC  NOSODS vs. HC |
| HDRS-17^a^  (non-sleep score, at screening) | | 7.5 (2.9) | 9.0 (4.0) | 2.6 (2.1) | <0.001 | | <0.001 | SODS vs. HC  NOSODS vs. HC |
| Sleep diary | |  |  |  |  | |  |  |
|  | Time in bed, min | 448.1 (59.0) | 482.0 (77.8) | 455.9 (43.2) | 0.600 | | 0.309 |  |
|  | Total sleep time, min | 258.2 (111.0) | 351.2 (95.0) | 425.7 (65.7) | <0.001 | | <0.001 | SODS vs. HC  SODS vs. NOSODS |
|  | Sleep latency, min | 70.0 (44.9) | 41.7 (40.5) | 12.2 (7.9) | <0.001 | | <0.001 | SODS vs. HC |
|  | Sleep efficiency, % | 52.7 (13.1) | 76.6 (9.0) | 94.7 (3.4) | <0.001 | | <0.001 | All pairs |
|  | WASO, min | 98.6 (67.4) | 20.3 (11.4) | 3.0 (2.6) | <0.001 | | <0.001 | SODS vs. HC  SODS vs. NOSODS |
| Polysomnographic data | |  |  |  |  | |  |  |
|  | TST, min | 351.4 (75.2) | 369.4 (80.2) | 397.8 (75.7) | 0.133 | | 0.272 |  |
|  | Sleep latency, min | 34.8 (55.3) | 48.0 (84.2) | 14.2 (15.5) | 0.135 | | 0.278 |  |
|  | Sleep efficiency, % | 76.5 (12.2) | 74.1 (16.0) | 85.8 (8.5) | 0.010 | | 0.032 | NOSODS vs. HC |
|  | WASO, min | 73.8 (39.7) | 89.6 (83.7) | 53.2 (34.9) | 0.112 | | 0.220 |  |
|  | AHI, number per hour | 5.9 (4.8) | 2.5 (2.8) | 4.0 (6.8) | 0.988 | | 0.238 |  |
|  | PLMSI, number per hour | 2.5 (8.0) | 0.8 (2.7) | 0.5 (1.3) | 0.462 | | 0.480 |  |
| Abbreviations: SODS, insomnia with subjective-objective discrepancy of sleep; NOSODS, insomnia without subjective-objective discrepancy of sleep; HC, healthy control; SD, standard deviation; ISI, Insomnia Severity Index; PSQI, Pittsburgh Sleep Quality Index; TST, total sleep time; PSAS, Pre-Sleep Arousal Scale; HDRS, Hamilton Depression Rating Scale; WASO, wake after sleep onset; AHI, Apnea–Hypopnea Index; PLMSI, periodic limb movements during sleep  Statistical tests were performed using Student’s *t-*test, analysis of variance, and chi-square test.  *^¶^* The post hoc analysis was performed using Bonferroni correction. ^a^HDRS-17 (non-sleep score): excluding the sleep items (4, 5, and 6) from total HDRS-17 score. | | | | | | | | |

| **Supplementary Table 2. Regression analysis of SOD, using TST and the BOLD response (*Ins* – *Neu*)** | | | | | | |
| --- | --- | --- | --- | --- | --- | --- |
|  |  | ***Ins* - *Neu*** | | | |  |
| **Explanatory variable** | **Region** | **Peak MNI coordinate (mm)** | | | **Peak Z score** | **Extents (K_E_)** |
|  |  | **x** | **y** | **z** |  |  |
| SOD, using TST | Left medial frontal cortex | -14 | 60 | 4 | 3.48 | 32 |
| Abbreviations: SOD, subjective-objective discrepancy; TST, total sleep time; BOLD, blood oxygen level dependent; *Ins*, insomnia item; *Neu*, neutral item; MNI, Montreal Neurological Institute | | | | | | |

**Supplementary Figures**

**Supplementary Figure 1. Increased BOLD signals of white matter to the *Ins* vs. *Neu* contrast among groups**


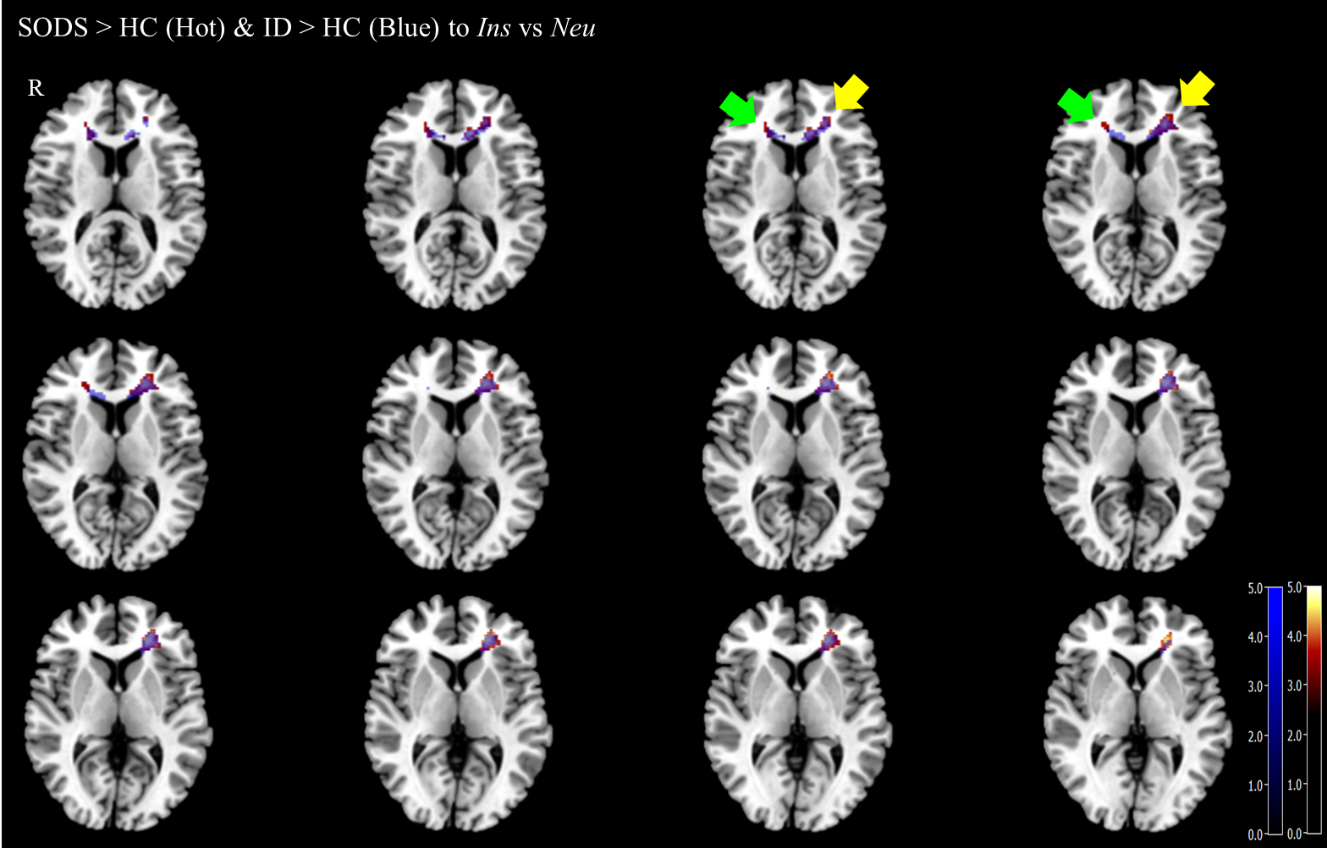


Brain areas showing increased BOLD signals in white matter to the contrast of *Ins* vs. *Neu* among groups (SODS vs. NOSODS vs. HC; ID vs. HC). Bilateral ACR and GCC showed increased BOLD signals in SODS and ID compared to the HC group (green and yellow arrows represent right and left ACR, respectively). The statistical threshold was voxel-wise uncorrected *p* < 0.001 with a cluster-wise FWE corrected *p* < 0.05 (141 voxels).

Abbreviations: BOLD, blood oxygen level dependent; *Ins*, insomnia item; *Neu*, neutral item; SODS, insomnia with subjective-objective discrepancy of sleep group; NOSODS, insomnia without subjective-objective discrepancy of sleep group; HC, healthy control; ID, insomnia disorder group (SODS + NOSODS); GCC, genu of corpus callosum; ACR, anterior corona radiate; FWE, family wise error.

**Supplementary Figure 2. Regression analysis of SOD, using TST and BOLD response (*Ins* – *Neu*)**


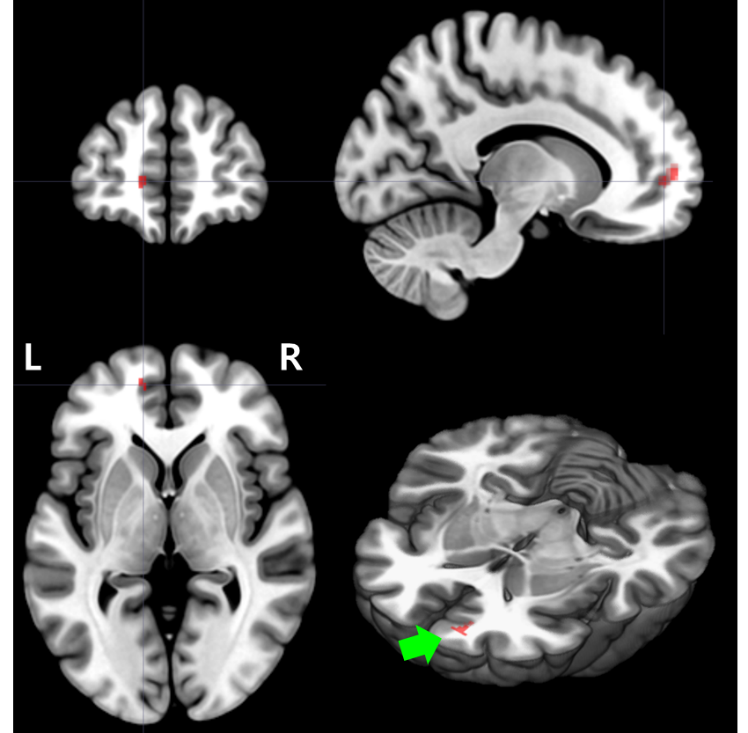


The regression analysis of SOD, using TST and the BOLD response showed an increased BOLD signal in the left medial frontal cortex using the criteria of voxel-wise uncorrected *p* < 0.001 with a cluster size of 30.

Abbreviations: SOD, subjective-objective discrepancy; TST, total sleep time; BOLD, blood oxygen level dependent; *Ins*, insomnia item; *Neu*, neutral item.

**Supplementary Figure 3.** **Task paradigm and analysis flow for the functional magnetic resonance imaging experiment**

(A) Task paradigm for the functional magnetic resonance imaging (fMRI) experiment. The fMRI experiment employed a block design featuring insomnia item (*Ins*), general item (*Gen*), and neutral item (*Neu*) trial. The function scans in each block has two periods, stimulus and response. In stimulus period, one of three items was presented for 6 sec according to the trial followed by cross sign and, consecutively, a response period was presented for 6 s. After finishing each block, the intertrial interval was jittered between 12 and 20 sec with a dot sign.

(B) Flow of the fMRI analysis


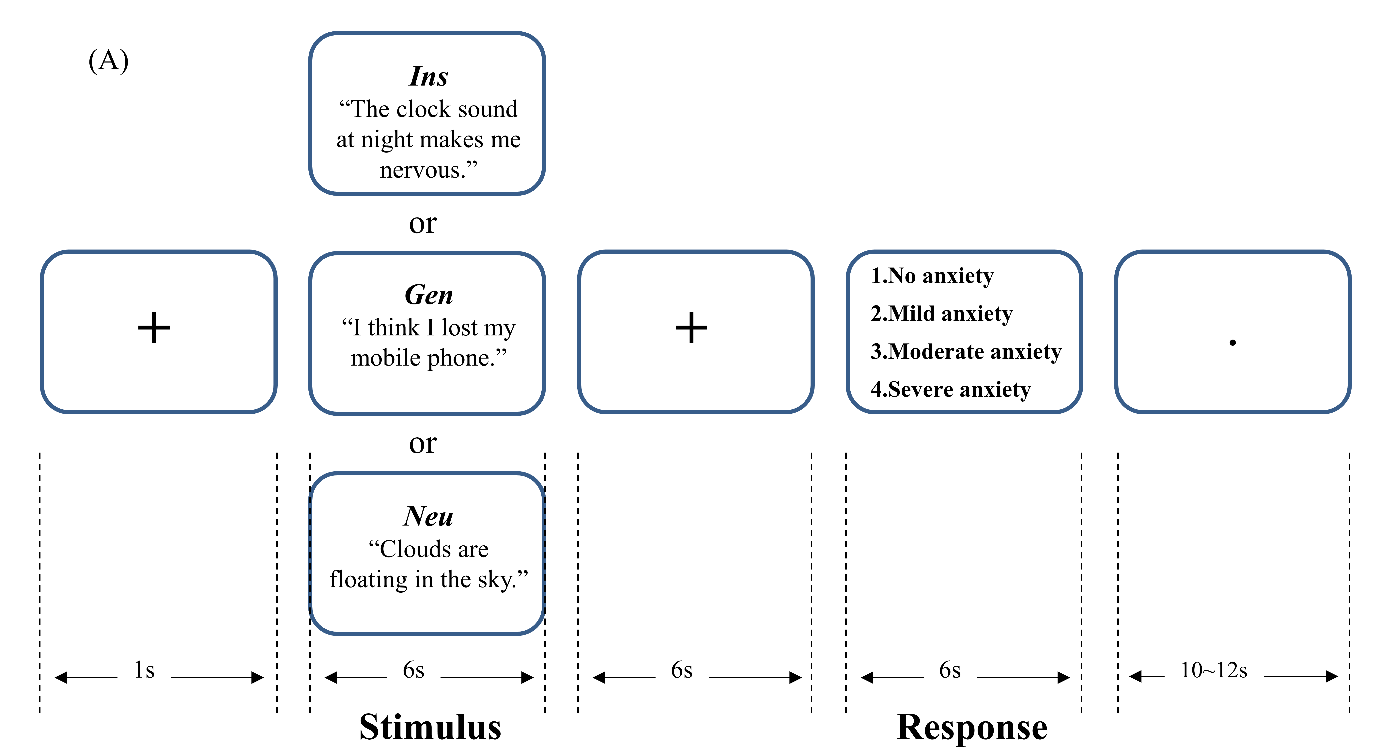


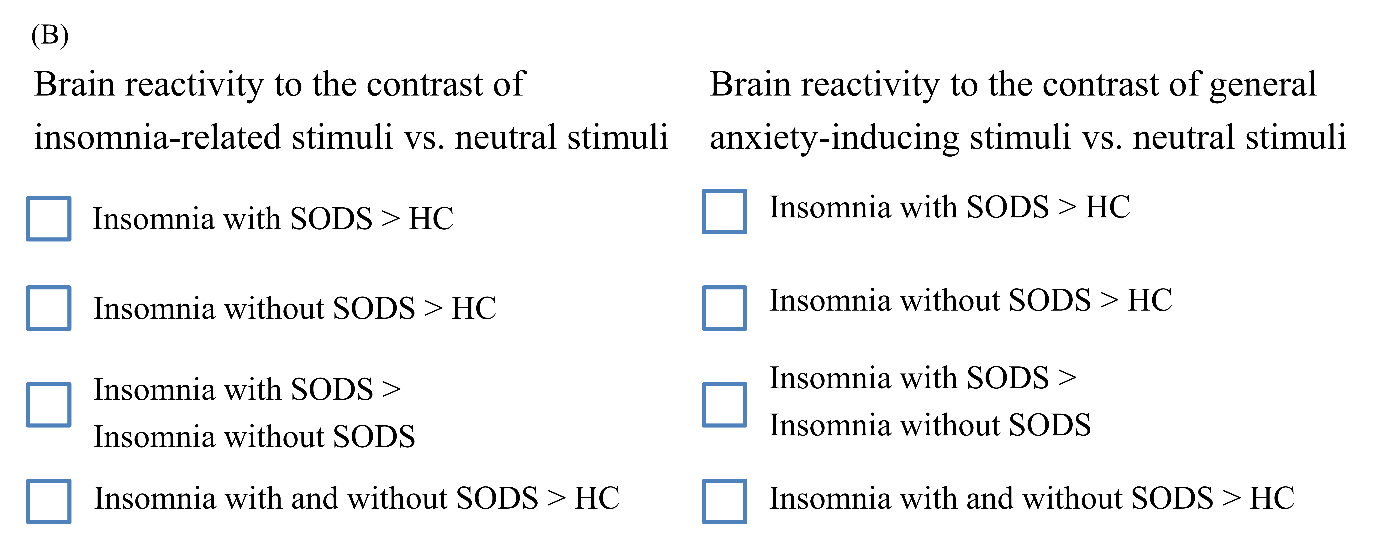

Supplement: Supplementary file 5 — Supplementary Information. [file 41598_2021_81219_MOESM5_ESM.docx]
